# Caring for cancer patients in the Covid pandemic: choosing between the devil and deep sea

**DOI:** 10.1186/s12957-020-02002-7

**Published:** 2020-08-22

**Authors:** Mainak Chakraborty, Manoj Pandey

**Affiliations:** Department of Surgical Oncology, Institute of Medical Sciences, Banaras Hindu University, Varanasi, 221005 India

**Keywords:** Covid, Cancer, CoV2, Coronavirus, Treatment, Chemotherapy, Surgery

## Abstract

**Background:**

Healthcare is an essential service at any time more so in the crisis like Covid. With increase in number of cases and mortality from Covid, the primary focus is shifted to the management of the Covid crisis and other health emergencies thus affecting normal health services and routine treatment of other diseases like cancer.

**Methods:**

This article reviews the published literature and guidelines on Covid and cancer and discusses them to optimize the care of cancer patients during Covid pandemic to improve treatment outcomes.

**Results:**

The results of the review of published literature show a twofold increase in probability of getting CoV2 infection by the cancer patients and a four-fold increase in chance of death. On the other hand, if left untreated a 20% increase in cancer death is expected. Data further show that none of the medicines like remdesivir, hydroxy chloroquin, dexamethasone, or azithromycin improves survival and response to Covid in cancer patients. Surgical results too show similar outcome before and after the pandemic though most of these report on highly selected patients populations.

**Conclusions:**

The Covid 2019 pandemic places cancer patients in a very difficult situation wherein if they seek treatment, they are exposing themselves to a risk of developing CoV2 infection and if they do not, the probability of dying without treatment increases. Hence, for them it is a choice between the devil and deep sea, and it is for the healthcare providers to triage patients and treat who cannot wait even though the data from the carefully selected cohort of patients show no increase in mortality or morbidity from treatment during Covid.

## Introduction

With the onset of Covid-19 pandemic, the situation for cancer patients has become a nightmare. Most of the patients who were on treatment in March had to miss out their further treatment due to lockdown and closure of hospitals and all modes of transport, both public and private. Those who developed cancer during lockdown too could not reach doctors for the same reason. Surgeries were postponed, radiotherapy was postponed, and there was uncertainty as far as chemotherapy and immunotherapy were concerned. Every society and organization came up with their own guidelines; however, none of these addressed the logistic problems that patients faced reaching the treatment centers. This lead to an increased pool of untreated patients; an analysis from England estimates additional 6270 deaths from cancer over next 12 months that is 20% increase proposed due to Covid pandemic [1].

Those who were willing to travel and take the risk of getting infected with Covid-19, too, faced challenges due to travel restrictions, though the Indian government issued special passes to allow travel for cancer treatment; however, the rider was that he or she has to travel with his own private transport and not more than two people were allowed in a four wheeler. This meant that apart from the driver, only the patient could travel without any caregiver, if the patient does not own the car and caregiver does not know how to drive. These passes were issued district or state wise as there was a ban on inter-district and interstate travel, so the patients coming to bigger institutes like ours faced special problems wherein despite having a travel pass, some of them had to pay fines. Most of our patients come from neighboring states of Bihar and Madhya Pradesh and even from West Bengal and Nepal.

Apart from that, at the beginning of the pandemic things were not clear and there was no data on the incidence and mortality from Covid-19 in cancer patients; further, though there were several guidelines [2–8], they were not based on evidence as the evidence did not exist at that time and it was not clear as to what treatment modalities are safe, giving rise to uncertainties in the mind of patients and health care workers. The testing facilities were limited, the availability of PPE was limited and these were prioritized for suspected cases and contacts, while other diseases were kept on hold or received low priority. This article reviews the current literature on various aspects of Covid and cancer.

## Methods

A review of literature was carried out using the pubmed for the articles published using the string “covid”[All Fields] AND (((((((((“cancer s”[All Fields] OR “cancerated”[All Fields]) OR “canceration”[All Fields]) OR “cancerization”[All Fields]) OR “cancerized”[All Fields]) OR “cancerous”[All Fields]) OR “neoplasms”[MeSH Terms]) OR “neoplasms”[All Fields]) OR “cancer”[All Fields]) OR “cancers”[All Fields]). Following filters were applied Clinical Study, Clinical Trial, Comparative Study, Controlled Clinical Trial, Meta-Analysis, Observational Study, Pragmatic Clinical Trial, Randomized Controlled Trial.

The articles that discussed the incidence, prevalence, mortality, treatment of cancer during Covid, or morbidity of treatment were included in the review. As the obtained data was very heterogeneous, no attempt at pooling or meta-analysis was done.

## Results

A total of 65 articles were extracted, the abstract of all the articles was read and after excluding reviews, 38 articles were included in this review including one meta-analysis. As for the prevalence, the initial data from a multicentric study comparing cancer with non-cancer patients infected with Covid found higher incidence of severe outcome in cancer compared to normal; the outcome was worse in lung cancer and metastatic disease [9]. A study evaluating fatality in 44,672 patients with Covid found a 2.9 times risk of death in cancer patients contracting Covid [10].

This fear and uncertainty had affected the quality of life and metal health of cancer patients, caregivers, and health care workers. There were no socializing, no outlets, no markets or movies or dining out, even the places of worships were closed. An Italian survey of young cancer patients showed that they feel more vulnerable to contract cancer, and risk of higher complications [11]. A higher burnout has been reported in healthcare workers working in Covid hospitals [12].

Over a period of time, things have started clearing up and preliminary data on incidence, severity, and complications of Covid-19 is now available. A retrospective analysis of 1590 patients across mainland China reported 16% incidence of severe cases [13]. Of these, 399 (25.1%) had at least one co-morbidity with hypertension (16.9%) being most prevalent followed by diabetes (8.2%). In this study, the cancer was seen only in 10 (0.9%) patients of whom 7 had severe illness. Another study from China on 1509 patients with Covid had 18 patients with cancer [14]. They too found higher severity in cancer patients; however, the number of cancer patients in this study too was very small. Similarly, the study of Chen et al. [15] had 10 patients in total of 1099 Covid-19 patients and only three of these had a severe disease. Similar results have been reported by others [16, 17].

A pooled meta-analysis of 11 studies reporting on Covid prevalence in cancer patients found a pooled prevalence of 2.0%; prevalence was higher at 3% in smaller series reporting on less than 100 patients [18]. Between March and June 2020, over 5400 patients were seen at our center, of which 237 undergoing surgery or the interventions were tested for Covid; of these, 9 patients were found to be positive (3.7%). The prevalence of cancer is high among Covid-19 patients in Europe; however, these do not seem to convert into higher mortality of cancer patients. A study from UK reporting on 226 deaths did not find increased mortality in cancer except in patients receiving chemotherapy [19]. However, a meta-analysis involving 46,499 patients that included 1776 cancer patients showed a significant increase in hazard of death in cancer patients infected with Covid-19 (HR 2.03) [20]. The study also showed increased admission to ICU (HR 1.56); however, when patients above the age of 65 were considered, no difference in death rate was observed [20]. This increase in cancer mortality in Covid-19 has been attributed to advance age, additional co-morbidities, diagnosis of lung cancer, use of chemotherapy etc. [19, 21–25].

Recent data from the USA, Canada, and Spain from the COVID-19 and Cancer Consortium (CCC19) database reported on 928 patients with cancer; of these, 121 (13%) patients had died [26]. Advance age, smoking, male gender, more than one co-morbidity, and ECOG more than 2 were found to be associated with increased mortality. These results and those detailed above clearly show that prevalence of Covid in cancer is higher and mortality in these patients too is higher, especially in patients with additional co-morbidities and active disease, and one needs to exercise full precaution while taking treatment decisions.

## Discussion

### Precautions taken at our center to prevent spread in cancer patients

As cancer patients are at a higher risk of contracting Covid-19 than the general population, special precautions are taken when patients are seen in the outpatient clinic (OPD) and surgery. No patient or the caregiver is allowed to enter the OPD without a face mask, all patients are screened for body temperature, and those found to have fever are immediately sent to Covid OPD. This is followed by filling up of a symptom checklist and questionnaire-based screening for all patients. Those with Covid symptoms are referred for RT-PCR testing before being allowed to see doctor and get the treatment. This is similar to all others coming to the hospital for treatment as there are no separate guidelines for cancer, apart from that strict hand hygiene is followed and PPE has to be worn by health care workers. Those planning to undergo procedures and surgery are screened for Covid-19 using RT-PCR testing. They are kept in the holding area and after being reported are shifted to wards. Any patient found positive is treated as per guidelines.

### Planned surgery

Elective surgeries for cancer patients are avoided if possible, this is usually a collective decision taken by the oncologist in consultation with the patient. If it is decided to do a surgery, a special Covid-19 consent is taken. All emergency surgeries are performed with proper written Covid consent and after Covid testing for even asymptomatic patients. Other than emergencies, patients who cannot wait for 4 weeks for surgery as the disease may progress and become inoperable are considered for surgery. Other than that, patients who were started on neoadjuvant therapy before the onset of Covid and have now completed neoadjuvant therapy are also candidates for surgery, as the opportunity of window period should not be lost. Any patient where the multidisciplinary team think can wait for 4 weeks is postponed. Patients undergoing surgery are reported to be at higher risk than those treated by radiation [9].

### Planned radiotherapy

In the beginning, all patients were postponed after discussion with patients or caregivers; however, as it is not possible to postpone radiation forever, selected patients are now being taken up for radiation. Patients are informed that whatever little evidence that is available suggest higher chances of Covid infection in this subset of patients. Recent guidelines have suggested omitting radiation for patients older than 65 years, using FAST and FAST forward, omitting boost, and hypofractionation [13, 14]. Recent articles also suggest modification of the delivery technique to reduce treatment time [27, 28].

### Immunosuppressive therapy

The current evidence does not support changing or withholding chemotherapy, targeted therapy, and immunotherapy in cancer patients. Some of the studies suggested stopping of immunotherapy for patients who have a complete response or prolonged response of more than 2 years [29]. A report on immune check point inhibitors suggested that as there is not much immune compromise and minimal hematological toxicities, they can be used with caution as patients are not devoid of its benefits [30, 31]. At our center, we have continued with chemotherapy and immunotherapy and have not found any increase in Covid-19 infection or mortality in this sub group.

### Stem cell transplantation

Current guidelines recommend delaying the planned allogenic stem cell transplant [32, 33]. This is a unique situation where the donor and recipient both are at risk and a careful decision making keeping all factors into account be made. No stem cell transplants took place at our center during this period.

### Coronavirus therapy in cancer

There is no evidence of benefit of using various therapies to treat Covid-19 in cancer patients. Remdesivir has been approved by the FDA for treatment of Covid cases under emergency use authorization. The CCC19 study failed to show any benefit of azithromycin, hydroxy chloroquine, alone or in combination; in fact, use of a combination of the two was found to be an independent factor predicting mortality with hazard ratio of 2.93, which was statistically significant [26]. There are occasional reports on the use of lopinavir/ritonavir [26, 34] in lung cancer patient with Covid. Another dataset from CCC19 study reported on 2186 patients treated with hydroxychloroquine (*n* = 538, 25%), azithromycin (*n* = 485, 22%), remdesivir (*n* = 124, 6%), high-dose corticosteroids (*n* = 109, 5%), tocilizumab (*n* = 94, 4%), and other therapy (*n* = 90, 4%) showed no benefit of any treatment. Apart from slight activity of remdesivir no other drug showed any benefit [26, 35]. Patients also received dexamethasone, aspirin, low-molecular weight heparin, and other anti-coagulants besides above treatments.

### Research on Covid-2019 and cancer

A recent bioinformatic study using the GEO database showed that angiotensin-converting enzyme 2 (ACE2) is elevated in uterine corpus endometrial carcinoma and renal papillary cell carcinoma. This increase also correlated with immune infiltrate present in the tumor; the authors suggested that these tumors may be more prone to Covid-19 infections [36]; however, the authors failed to present any evidence to suggest this hypothesis. Similar bioinformatics study by Fu et al. also showed similar results and they suggested that testes may also get affected by Covid-19 infections [37]. Other tumors that may be affected are pancreas and colon [38]. However, all these studies are based on GEO databases and there is no experimental evidence presented till date.

## Conclusions

The literature on cancer and Covid is fast expanding with more and more information pouring in. The literature so far is clear in indicating that cancer patients are at high risk of developing Covid and when developed, the severity and mortality is higher than the normal population. The recent data also suggests a possible role of remdesivir in the treatment of Covid-19 in cancer patients; however, the evidence to support this is very weak. This absence of conclusive evidences has led to development of strategies to treat cancer safely; most of these are based on reducing the hospital visits and avoiding immune compromise. The evidence is still evolving and more practice changes are expected in the days to come and that may continue even in post Covid era.

## Data Availability

Not applicable
